# Supplementary material for: Polyketide synthase gene clusters in the ericoid mycorrhizal fungus Oidiodendron maius: genome-wide prediction, expression profiling, and functional characterization of knockout mutants
Source: Front Plant Sci. 2026 Sep 16;17:1924430. doi: 10.3389/fpls.2026.1924430 (PMC13623903; doi:10.3389/fpls.2026.1924430)
Supplement: Supplementary file 1 [file SupplementaryFile1.zip › Supplementary Materials/Supplementary Folder 1.zip!/Oidma1_AssemblyScaffolds_Repeatmasked/knownclusterblast/cluster34/jgi.p_Oidma1_131498_mibig_hits.html]

| MIBiG Protein | Description | MIBiG Cluster | MiBiG Product | % ID | % Coverage | BLAST Score | E-value |
| --- | --- | --- | --- | --- | --- | --- | --- |
| ADI24956.1 | GsfD | BGC0000070 | Griseofulvin\_biosynthetic\_gene\_cluster | 28 | 91.7274939173 | 168 | 2.2e-41 |
| CDM36722.1 | O-methyltransferase,\_caffeic\_acid-type | BGC0001360 | Mycophenolic\_acid\_biosynthetic\_gene\_cluster | 27 | 88.5644768856 | 134 | 2e-31 |
| BAE95598.1 | putative\_O-methyltransferase | BGC0000703 | Kanamycin\_biosynthetic\_gene\_cluster | 28 | 74.695863747 | 81 | 3.5e-15 |
| CAF60515.1 | putative\_O-methyltransferase | BGC0000704 | Kanamycin\_biosynthetic\_gene\_cluster | 28 | 74.695863747 | 81 | 3.5e-15 |
| CAF31570.1 | putative\_O-methyltransferase | BGC0000705 | Kanamycin\_biosynthetic\_gene\_cluster | 28 | 74.695863747 | 81 | 3.5e-15 |
| AGY49596.1 | PbtM4 | BGC0001155 | GE2270\_biosynthetic\_gene\_cluster | 35 | 35.0364963504 | 78 | 1.7e-14 |
| ACS83775.1 | O-methyl\_transferase | BGC0000604 | GE2270\_biosynthetic\_gene\_cluster | 35 | 35.0364963504 | 76 | 1.1e-13 |
| AAP69580.1 | putative\_O-methyltransferase | BGC0000226 | Gilvocarcin\_biosynthetic\_gene\_cluster | 32 | 38.9294403893 | 73 | 7.3e-13 |
| EAL89337.1 | O-methyltransferase,\_putative | BGC0001403 | Trypacidin\_biosynthetic\_gene\_cluster | 31 | 42.5790754258 | 69 | 1.1e-11 |
